# FRAILTY-FOCUSED COMMUNICATION (FCOM) FOR OLDER ADULTS

**DOI:** 10.1093/geroni/igac059.881

**Published:** 2022-12-20

**Authors:** Cathy Maxwell, Sally Miller, Deborah Lee

**Affiliations:** Vanderbilt University, Hermitage, Tennessee, United States; Vanderbilt University, Nashville, Tennessee, United States; Middle Tennessee State University, Murfreesboro, Tennessee, United States

## Abstract

Frailty-focused communication (FCOM) equips older adults with a better understanding of factors and biological mechanisms related to decline, frailty, and eventual end-of-life, while also empowering them to engage in proactive measures for healthy aging in 8 domains (physical activity, nutrition, safety, mind/body health, relationships/community, sleep/rest, finances, health care decision-making). We aimed to develop and test: 1) an educational tool targeted to older adults, and 2) a FCOM workshop to train health care clinicians on how to explain the concept of frailty and facilitate goal-setting for proactive aging. We tested the tool and workshop in 2 prospective observational studies among community-dwelling older adults (N= 126) and clinicians (N=29) who provide care to older adults. Testing of the FCOM tool revealed that content on bioenergetics of aging increased motivation to make a plan for proactive aging (N=60, 68%), particularly among adults age 55-64 as compared to other age groups (p=.024). Testing of the training workshop demonstrated increased knowledge of frailty (pre score: 5.8, post score: 7.6; d= .55, p=.006) and increased self-reported competency with patient/clinician interaction (pre score: 33.2, post score: 42.0; d=.63, p=.001). Post course evaluations regarding understanding of best practice empathic skills was rated 3.6 (range: 1-4). FCOM promotes awareness of the importance of measures to mitigate development of chronic conditions and frailty, and provides a resource for clinicians in multiple settings to engage in conversations with older adults while physiologic compensatory mechanisms are still intact. FCOM discussions can facilitate a shift from disease-oriented discussion to patient-centered, holistic care.

